# 566 Burn Camp Volunteers Exhibit High Levels of Emotional Intelligence; Attributes to Consider for Volunteer Recruitment

**DOI:** 10.1093/jbcr/irae036.200

**Published:** 2024-04-17

**Authors:** Ruth Brubaker Rimmer, Curt C Bay, Emile T Kalil, Daniel W Chacon, Tess Robaina, Kevin N Foster

**Affiliations:** Ariaona Burn Center, Phoenix Arcadia, AZ; A.T. Still University, Mesa, AZ; University of Maryland School of Medicine, Dept. PTRS, Highland, MD; Alisa Ann Ruch Burn Foundation, San Francisco, CA; Arizona Burn Foundation, Phoenix, AZ; Arizona Burn Center Valleywise Health, Phoenix, AZ; Ariaona Burn Center, Phoenix Arcadia, AZ; A.T. Still University, Mesa, AZ; University of Maryland School of Medicine, Dept. PTRS, Highland, MD; Alisa Ann Ruch Burn Foundation, San Francisco, CA; Arizona Burn Foundation, Phoenix, AZ; Arizona Burn Center Valleywise Health, Phoenix, AZ; Ariaona Burn Center, Phoenix Arcadia, AZ; A.T. Still University, Mesa, AZ; University of Maryland School of Medicine, Dept. PTRS, Highland, MD; Alisa Ann Ruch Burn Foundation, San Francisco, CA; Arizona Burn Foundation, Phoenix, AZ; Arizona Burn Center Valleywise Health, Phoenix, AZ; Ariaona Burn Center, Phoenix Arcadia, AZ; A.T. Still University, Mesa, AZ; University of Maryland School of Medicine, Dept. PTRS, Highland, MD; Alisa Ann Ruch Burn Foundation, San Francisco, CA; Arizona Burn Foundation, Phoenix, AZ; Arizona Burn Center Valleywise Health, Phoenix, AZ; Ariaona Burn Center, Phoenix Arcadia, AZ; A.T. Still University, Mesa, AZ; University of Maryland School of Medicine, Dept. PTRS, Highland, MD; Alisa Ann Ruch Burn Foundation, San Francisco, CA; Arizona Burn Foundation, Phoenix, AZ; Arizona Burn Center Valleywise Health, Phoenix, AZ; Ariaona Burn Center, Phoenix Arcadia, AZ; A.T. Still University, Mesa, AZ; University of Maryland School of Medicine, Dept. PTRS, Highland, MD; Alisa Ann Ruch Burn Foundation, San Francisco, CA; Arizona Burn Foundation, Phoenix, AZ; Arizona Burn Center Valleywise Health, Phoenix, AZ

## Abstract

**Introduction:**

Volunteers are the lifeblood of burn camp. However, there is a paucity of information regarding the individuals who donate significant time and resources to the camp process. This study sought to assess participant demographics and to measure their emotional intelligence. Higher emotional intelligence (EI) is associated with the ability to monitor and label one’s own and others' emotions and use these skills to pay better attention, be more engaged and more empathetic to others while developing positive relationships; all skills important for working successfully and effectively with burn-injured youth.

**Methods:**

Burn camp volunteers from 3 U.S. burn camps completed the Assessing Emotions Scale (AES), a 33-item valid self-report screen measuring Emotional Intelligence (EI). Respondents rate themselves using a 5-point Likert scale ranging from 5 (strongly agree) to 1 (strongly disagree). A total score is calculated from the sum of all items. A higher score indicates a higher level of EI.

**Results:**

Participants included 119 adult volunteers, female (n=64), and male (n=53). By ethnicity, counselors were Caucasian (75%), Hispanic (13%), African American (3%), and Asian (3%), with the majority in the 20-45-year age range (76%). Volunteers’ professions included firefighters (24%), medical professionals (16%), educators (5%), business (11%), and other (49%). Many (34%) reported being burn survivors, and 26% reporting visible scarring. Female and male participants scored significantly higher than normative means - females 130.05 vs. norm of 121.1. (p< 0.001) and males 129.4 vs. norm of 107.01 (p< 0.001). Burn survivors and Hispanics both reported notably high mean scores of 132. Educators had the highest mean at 137.

**Conclusions:**

It appears that the participating burn camps are doing a good job of recruiting volunteers with high emotional intelligence. This is important because it indicates the participant characteristics include optimism, impulse control, and greater empathic perspective-taking. Of note, the majority of participating counselors were Caucasian, as compared to only 33% of campers.

**Applicability of Research to Practice:**

A concerted effort for the continued recruitment of counselors exhibiting high EI attributes is advised. EI testing may be a beneficial screening tool for volunteer applicants as the competition for burn camp volunteer slots.

